# How Does the Gradient Measure of the Lung SBRT Treatment Plan Depend on the Tumor Volume and Shape?

**DOI:** 10.3389/fonc.2021.781302

**Published:** 2021-11-15

**Authors:** Yanhua Duan, Yang Lin, Hao Wang, Bodong Kang, Aihui Feng, Kui Ma, Hua Chen, Ying Huang, Hengle Gu, Yan Shao, Tao Zhou, Qing Kong, Zhiyong Xu

**Affiliations:** ^1^ Department of Radiation Oncology, Shanghai Chest Hospital, Shanghai Jiao Tong University, Shanghai, China; ^2^ Pekoe Team, MIM Software Inc., Cleveland, OH, United States; ^3^ Clinical Helpdesk, Varian Medical Systems, Beijing, China; ^4^ Shandong Cancer Hospital and Institute, Shandong First Medical University and Shandong Academy of Medical Sciences, Jinan, China; ^5^ Institute of Modern Physics, Fudan University, Shanghai, China

**Keywords:** SBRT, lung cancer, radiotherapy, gradient measure, volume, shape

## Abstract

**Purpose:**

Gradient measure (GM) is a critical index related to normal tissue sparing in radiosurgery. This study aims to describe the dependence of GM on target volume and target shape for lung stereotactic body radiation therapy (SBRT) treatment plans.

**Methods:**

A total of 307 peripheral and 119 central lung SBRT treatment plans were enrolled for this study. A least-squares regression was used for data analysis. First, the equations with different functional forms were established to determine the dependence of GM on a univariaty (V_P_ or Sp) and bivariaty (V_P_ and Sp), respectively. Then, the correlation coefficients and p-values of variables for all equations were compared and analyzed to determine the dependence of GM on PTV volume (VP) and sphericity (Sp).

**Results:**

The power equations had the highest coefficient of determination (R^2^) in the dependence results of GM on univariate V_P_. The equations were $\text{GM}=0.674{\text{V}}_{P}^{0.178}$ and $\text{GM}=0.660{\text{V}}_{P}^{0.185}$ for peripheral and central lesions, respectively. On the other hand, the R^2^ of all functional forms were less than 0.25 when the relationship of GM versus univariate Sp was analyzed. Similarly, the power equation also obtained the highest R^2^ in bivariaty V_P_ and Sp analysis, whether for central or peripheral. However, the R^2^ of the bivariate equations were not improved compared with those of univariate equations. Moreover, the p-values of the variable Sp were greater than 0.05.

**Conclusions:**

The GM of the lung SBRT plan is shape-independent and volume-dependent. The dependence of GM on PTV volume for peripheral and central lung cancer can be described by two different power equations. The results of this study can be used as a potential tool to assist dosimetric quality control during the radiosurgery process.

## Introduction

As a technique that has been widely employed, stereotactic body radiotherapy (SBRT) can be used to deliver high fractional dose in few fractions. Compared with traditional radiotherapy, SBRT provides better efficacy, lower toxicity, and shorter treatment duration (1, 2). Clinical evidence and studies have shown that the therapeutic effect of early non-small cell lung cancer (NSCLC) patients treated with SBRT is similar to or even better than that of surgery (1, 2) and that SBRT has become a major alternative therapy for patients with NSCLC who are unsuitable or unwilling to undergo surgery (3–5).

In order to achieve a therapeutic effect similar to surgery, a highly conformal SBRT treatment plan needs to give the tumor high-dose precise ablation while minimizing organs at risk (OARs) damage, which requires a sharp dose gradient nearly isotropically around the target (1, 2, 6–8). In clinical practice, the targets of different patients are various, and the dose gradient largely depends on the individualized geometric characteristics of the target (7, 9–13).

Some scholars have recently studied the dependence of the dose gradient on the target volume for SBRT plans (7, 14). Those studies lay a foundation for the dependence of dose gradient on target geometry in lung SBRT plan. However, most studies focused on the relationship between dose gradient and target volume, while the impact of target shape on the dose fall-off is still unclear. In addition, lung cancer patients treated with SBRT include peripheral and central types. Due to the significant difference in tumor anatomical location between those two types, the factors considered in the planning process are also different. It is unclear whether this leads to the dependence difference of dose gradient on target volume and shape between two types of lung cancer. Although RTOG has used the same criteria for R50%, which is defined as the ratio of 50% prescription isodose volume to the PTV volume characterizing the dose fall-off for both peripheral and central lesions, the difference in the location of the two types of tumors results in their different dose gradients. Therefore, it is necessary to investigate the dependence of dose gradient on target volume and shape, respectively, for peripheral and central lung SBRT plans.

In this study, a large number of clinically acceptable peripheral and central lung SBRT plans were used to analyze the dependence of dose gradient on target volume and shape. The results are to determine a definite relationship between the dose gradient and target geometry for lung SBRT plans and provide a possible tool for the prediction of dose gradient before the planning process or the quality review after optimization of a lung SBRT plan.

## Materials and Methods

The approved lung SBRT treatment plans in Shanghai Chest Hospital were retrospectively selected. When the study began, all selected patients signed informed consents and completed radiotherapy. Ethical standards and patients’ confidentiality were ensured and in line with regulations of the local institutional review board and data safety laws. This study was approved by the Ethics Committee of Shanghai Chest Hospital (the committee’s reference Number: KS1863).

### Target Delineation and Treatment Planning

Targets and OARs were delineated on a MIM Maestro Station (MIM Vista Corp, Cleveland, US-OH) based on four-dimensional CT (4DCT) by radiation oncologists. All structures were reviewed and approved by an experienced radiation oncologist before being used for planning design. All the treatment plans were planned on the average 4DCT image using the Pinnacle Treatment Planning System (TPS) (V9.10, Philips Radiation Oncology Systems, Fitchburg, WI, USA) for an Edge™ linear accelerator (Varian Medical Systems, Palo Alto, CA) equipped with a high-definition 120 multileaf collimator (MLC). The included treatment plans ranged from three to eight fractions, and the planning method was similar to our previous research (15). In short, treatments were planned following the guidelines of RTOG 0813 (16) or 0915 (17) depending on its tumor size, the patient’s physical condition, and location, which employed the IMRT technique with 10 or more 6MV fields. Collimator and couch angles were adjusted according to the individual situation. The collapsed cone convolution (CCC) algorithm was used for dose calculation with a calculation resolution of 1.0 mm.

### Data Extraction

This study analyzed the dependence of gradient measure (GM) (10) on PTV volume (V_P_) and sphericity (Sp).

The Eclipse (Varian, Palo Alto, CA) TPS reports GM, which is defined as the difference, in centimeters, of the equivalent sphere radii of the 50% and 100% prescription isodose line volumes (7, 10). This metric can quickly assess the dose gradient and has become a helpful tool for evaluating lung SBRT plans.

GM was computed as (8, 10, 18)


(1) $$GM=\sqrt[{3}]{\frac{3V_{50\%Rx}}{4\pi }}-\sqrt[{3}]{\frac{3V_{Rx}}{4\pi }}$$

where V_50%Rx_ and V_Rx_ are the volumes receiving a dose equal to or greater than 50% and 100% prescription dose, respectively.

Sphericity is a parameter that characterizes the shape of a three-dimensional structure. It is defined as the quotient of the surface area of a sphere and the surface area of a structure with the same volume. Sp ranges from 0 to 1, where Sp = 1 indicates a sphere.

Sp was calculated as


(2) $$S_{p}=\frac{4\pi {\left(\frac{3V_{P}}{4\pi }\right)}^{\frac{2}{3}}}{S_{PTV}}$$

where S_PTV_ is the surface area of PTV.

It can be seen from Equations 1 and 2 that the original data used for analysis include V_P_, 50% and 100% prescription isodose volumes, and Sp. First, a prewritten script in Pinnacle TPS was used to convert 50% and 100% prescription isodose lines to structures, and those structures were imported into MIM Maestro Station together with PTV contour. Then, a workflow embedded in the MIM Maestro station was used to calculate and extract the above four data.

### Data Analysis

The dependence of GM on V_P_ and Sp was analyzed using the least-squares regression (7). All analyses were performed using SPSS 22.0 (SPSS Inc., Armonk, NY).

Firstly, curve estimation was used to analyze the dependence of GM on a univariaty (V_P_ or Sp) for peripheral and central lung SBRT plans. The curve fitting included linear, logarithmic, exponential, power, and logistic functional forms. Then, the fitting equations of GM on bivariaty (V_P_ and Sp) were established. The regression equations include linear (Eq. 3), nonlinear sum (Eq. 4), logarithmic (Eq. 5), exponential (Eq. 6), and power (Eq. 7) functional forms.


(3) $$GM=aV_{P}+cSp+e$$

(4) $$GM=aV_{P}^{b}+cSp^{d}+e$$

(5) $$GM=\log_{a}V_{P}^{b}Sp^{c}+d$$

(6) $$GM=ab^{V_{P}}c^{Sp}+d$$

(7) $$GM=aV_{P}^{b}Sp^{c}+d$$

The coefficient of determination (R^2^) is the standard metric for evaluating the fitting goodness between model simulations and observations. Generally, the fitting model can be considered acceptable if R^2^ is equal to or above 0.5 (19). The p-value of the variable can reflect the reliability of a fitting equation. The contribution of a variable to an equation is considered reliable when p<0.05.

### Result Verification

In order to verify the accuracy of the final fitting equation, we use an independent external verification set to test the results to obtain the error between the calculated GM and the actual GM. The validation set included 100 peripheral lung cancer SBRT plans and 40 central lung cancer SBRT plans.

## Results

### Details of the Enrolled Cases

**Figure 1:**
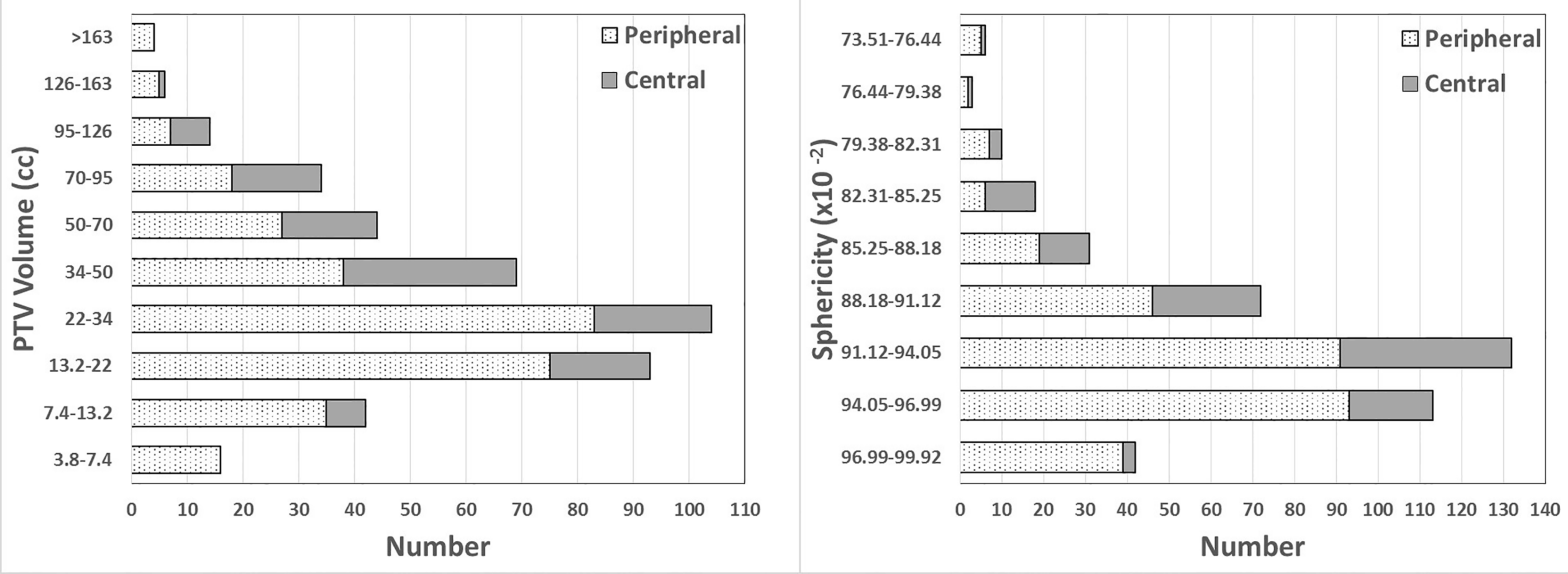
Number distribution of PTV volumes and sphericity in this study. PTV volume is presented using the RTOG 0813 and 0915 volume bins. Sphericity is shown using bins equally spaced according to the sphericity range in this study.

A total of 426 lung SBRT plans in our center from May 2018 to June 2021 were enrolled for this study, including 307 (72%) peripheral and 119 (28%) central. Central was defined as being within a 2-cm radius of the airway or mediastinal pleura (7). For peripheral, V_P_ ranged from 4.79 to 261.77 cc, and Sp ranged from 74.86 x 10^-2^ to 99.92 x 10^-2^. For central, V_P_ ranged from 7.76 to 144.56 cc, and Sp ranged from 73.51 x 10^-2^ to 97.31 x 10^-2^. The number distribution of the V_P_ and Sp for peripheral and central lesions is shown in **Figure 1**. The dose constraints to the targets and OARs met the proposal of RTOG 0813 (16) or 0915 (17) guidelines in all plans. Averages of the treatment plan data binned using V_P_ bins from RTOG 0813 and 0915 are listed in **Table 1**. **Table 1** also lists the actual and analytic GM values of each group for comparison, and the two results were similar. In order to refer to RTOG metrics, we also listed the planned R50% value in **Table 1**.

**Table 1:** Averages of the data for all lung SBRT treatment plans.

Volume bin (cc)	Tumor type	N	V_P_ (cc)	Sp (x10^-2^)	Actual GM (cm)	Analytic GM (cm)	R50%	CI	Rx dose (Gy)	Fractions	IMRT Factor
3.8-7.4	Peripheral	15	6.25	98.20	0.92	0.93	6.03	0.81	48.67	4.13	2.24
	Central	0	–	–	–			–	–	–	–
7.4-13.2	Peripheral	35	10.32	95.81	1.04	1.02	5.62	0.84	48.69	4.31	2.12
	Central	7	9.67	94.77	1.02	1.00	5.48	0.85	55.71	7.29	1.99
13.2-22	Peripheral	75	18.37	93.91	1.13	1.13	4.80	0.86	47.52	4.59	2.08
	Central	18	17.57	93.19	1.11	1.12	4.86	0.85	56.33	7.44	2.03
22-34	Peripheral	83	26.20	92.31	1.20	1.20	4.47	0.87	47.35	4.64	2.03
	Central	21	28.68	92.14	1.20	1.23	4.34	0.87	54.00	7.05	1.94
34-50	Peripheral	38	41.51	90.83	1.31	1.31	4.21	0.86	44.42	4.63	2.02
	Central	31	42.13	90.28	1.34	1.32	4.31	0.81	53.81	7.03	1.90
50-70	Peripheral	27	58.49	89.79	1.39	1.39	3.86	0.85	41.63	4.59	1.91
	Central	17	59.94	89.21	1.44	1.41	4.37	0.86	52.24	6.82	1.93
70-95	Peripheral	18	79.01	89.22	1.55	1.47	4.06	0.88	36.78	4.33	1.87
	Central	16	81.75	87.75	1.55	1.49	3.94	0.87	54.00	7.06	2.02
95-126	Peripheral	7	108.61	90.51	1.61	1.55	3.65	0.88	37.71	4.43	1.87
	Central	7	105.03	87.58	1.47	1.56	3.89	0.87	50.57	6.57	1.89
126-163	Peripheral	5	146.95	86.07	1.58	1.64	3.49	0.87	38.00	4.60	1.71
	Central	2	188.56	87.02	1.63	1.73	3.14	0.90	42.00	5.5	1.73
>163	Peripheral	4	212.09	83.94	1.58	1.74	2.92	0.88	27.00	3.50	2.14
	Central	0	–	–	–			–	–	–	–

IMRT factor is the quotient of fractional monitor units and fractional dose in cGy.

N, the number of cases; V_P_, PTV volume; Sp, sphericity; GM, gradient measure; CI, conformity index (the quotient of the PTV volume receiving the prescription dose and the PTV volume); Rx dose, prescription dose.

### Results of Univariate Analysis

**Table 2** lists the R^2^ of different dependence equations of GM on V_P_ and the p values of the independent variable. The power equation had the highest R^2^ in all functional forms (p < 0.001) for two types of lung SBRT plans. The equations of GM versus V_P_ were Equations 8 and 9 for peripheral and central lesions, respectively.


(8) $$GM=0.674V_{P}^{0.178}$$

**Table 2:** Fitting results of GM versus a univariaty (V_P_, Sp) using different functional forms.

Equation	Peripheral				Central			
	Vp		Sp		Vp		Sp	
	R^2^	p-V_P_	R^2^	p-Sp	R^2^	p-V_P_	R^2^	p-Sp
Linear	0.500	p<0.001	0.229	p<0.001	0.388	p<0.001	0.150	p<0.001
Logarithmic	0.667	p<0.001	0.222	p<0.001	0.521	p<0.001	0.143	p<0.001
Exponential	0.467	p<0.001	0.239	p<0.001	0.378	p<0.001	0.152	p<0.001
Power	0.675	p<0.001	0.230	p<0.001	0.526	p<0.001	0.146	p<0.001

V_P_, PTV volume; Sp, sphericity; GM, gradient measure.

with a standard error of 0.016 and 0.007 for the two parameters and an R^2^ value of 0.675.


(9) $$GM=0.660V_{P}^{0.185}$$

with a standard error of 0.040 and 0.016 for the two parameters and an R^2^ value of 0.526.

The improved R^2^ in Equation 8 shows that it can explain a greater percent of the random variation of peripheral lesions’ GM than Equation 9 can explain that of central lesions.

**Figure 2:**
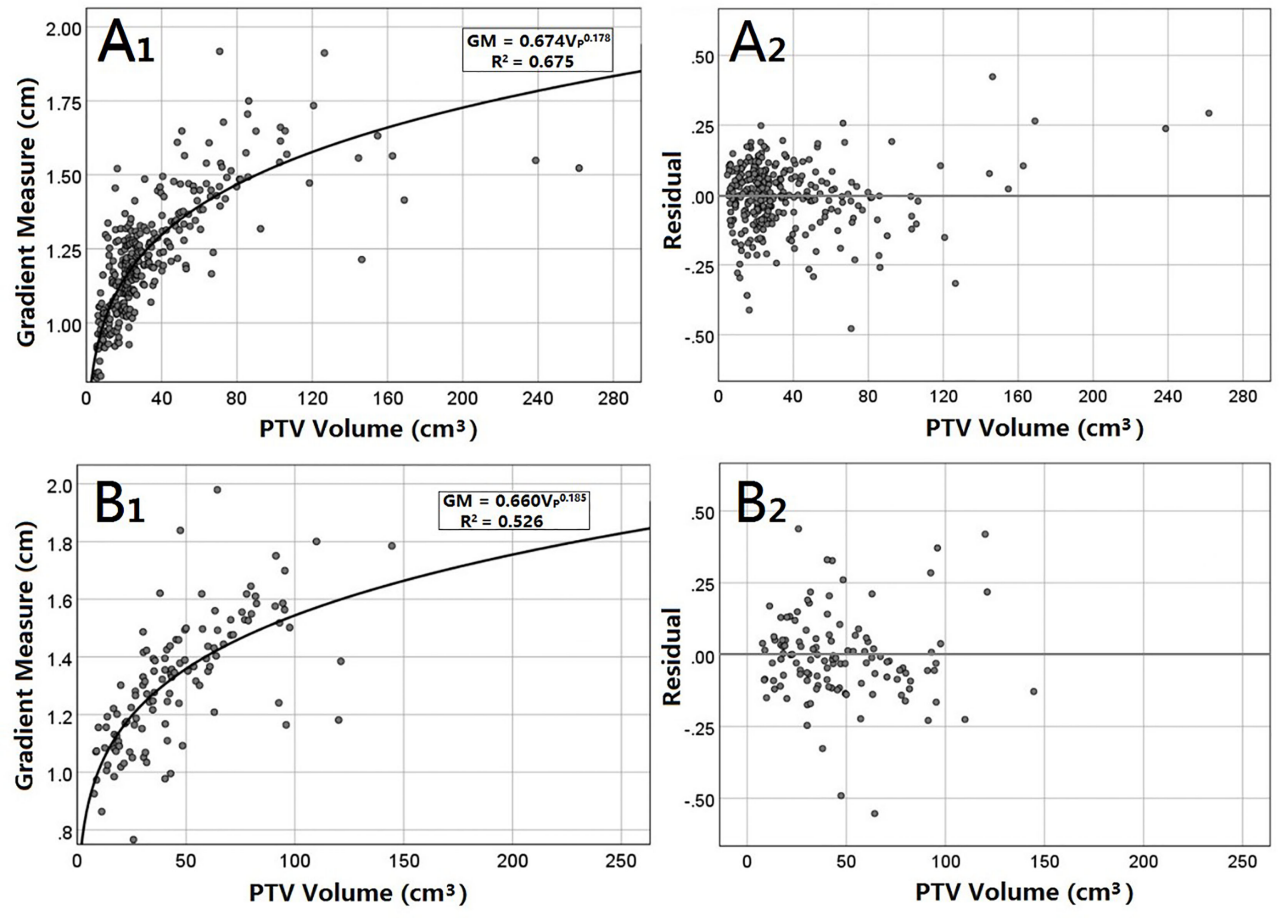
GM versus the V_P_ for peripheral **(A)** and central **(B)** lung SBRT plans. The least-squares fit of the power equations **(A_1_, B_1_)** is presented along with R^2^. In addition, residuals of the analytic GM minus the clinical GM are also presented **(B_1_, B_2_)**.

**Figure 2** presents GM versus VP scatter plots, including figures of power equations and their residuals for peripheral and central lesions. For peripheral lesions, the residuals appear to be nearly randomly distributed. Most of them are within 0.25 cm. Equation 8 predicted a lower GM in 149 cases (48.53%) and a greater GM in 158 cases (51.47%) than in the clinical plans. When V_P_ was greater than 125 cm^3^, Equation 8 consistently analyzed a higher GM than the actual value. For central lesions, the distribution of residuals also seems random. Equation 9 analyzed a lower GM in 67 cases (56.30%) and a greater GM in 52 cases (43.69%) than what was achieved clinically.

**Figure 3:**
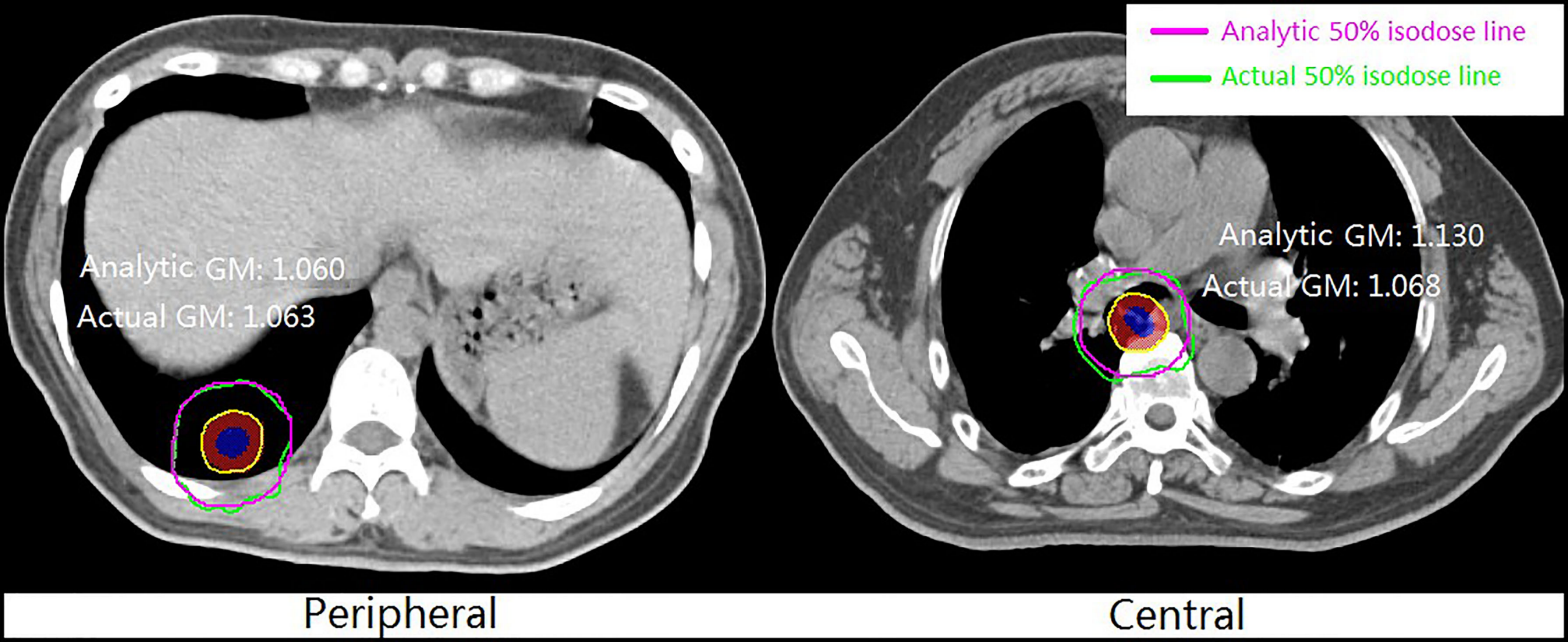
Example of analytic and clinical 50% isodose lines for peripheral and central lung SBRT plans.

**Figure 3** shows an example of the analytic and clinical 50% isodose lines for peripheral and central lung SBRT plans. The analytic 50% isodose lines were generated by GM calculated using Equations 8 and 9. It can be seen from the figure that the analytic and clinical dose gradients are in good agreement, especially for the peripheral lesion.

The R^2^ of different fitting equations of GM versus Sp and p values of variable Sp are also tabulated in **Table 2**. Among them, the exponential equation had the highest R^2^, but less than 0.25 (0.239 for peripheral and 0.152 for central) (p < 0.001). There was a weak correlation between the analytic and clinical results. The fitting equation was unacceptable, which signifies that the GM has little dependence on the shape variable Sp for both peripheral and central lung SBRT plans.

### Results of Bivariate Analysis

**Table 3** presents the R^2^ of different fitting equations and the p-value of two independent variables in the dependence analysis of GM on bivariaty V_P_ and Sp. The power equation obtained the highest R^2^, and the expressions of peripheral and central lesions are Equations 9 and 10, respectively.


(9) $$GM=0.676V_{P}^{0.175}Sp^{-0.069}$$

**Table 3:** Fitting results of GM on bivariaty (V_P_ and Sp) for different equations.

Equation		Peripheral			Central	
	R^2^	p-V_P_	p-Sp	R^2^	p-V_P_	p-Sp
Linear	0.523	p<0.001	p<0.001	0.400	p<0.001	p<0.001
Nonlinear sum	0.668	p<0.001	0.717	0.393	p<0.001	0.324
Logarithmic	0.667	p<0.001	0.759	0.521	p<0.001	0.630
Exponential	0.498	p<0.001	p<0.001	0.521	p<0.001	p<0.001
Power	0.675	p<0.001	0.590	0.527	p<0.001	0.494

with standard errors of 0.011, 0.009, and 0.128 for the three parameters.


(10) $$GM=0.662V_{P}^{0.181}Sp^{-0.100}$$

with standard errors of 0.027, 0.019, and 0.259 for the three parameters.

The R^2^ of bivariate power equations for peripheral and central lesions was 0.675 and 0.527, respectively, almost equal to fitting results with V_P_ as the independent variable (0.675 and 0.526). Compared with the equation of GM versus V_P_, the bivariate equation does not improve the fitting goodness. In addition, the p-value of the power equations for peripheral and central lesions were less than 0.001, while the p-values of the variable Sp were greater than 0.05 (0.590 for peripheral and 0.494 for central), indicating that the original hypothesis that the variable Sp was zero could not be rejected. In the bivariate equation, Sp could hardly explain the GM, and these equations were unreliable.

### Results of the Equation Verification

Using external independent validation sets, **Table 4** lists the maximum, minimum, mean error, and standard deviation between analytical GM and actual GM for peripheral and central lung cancer. The mean absolute errors were 0.017 and 0.023 cm for peripheral and central lung cancer, respectively. We can see that both mean absolute errors are less than 0.03 cm, and the standard deviation was slight (about 0.01 cm).

**Table 4:** Absolute error of analytical and actual GM.

GM (cm)	Peripheral	Central
Maximum	0.040	0.047
Minimum	<0.001	<0.001
Mean	0.017	0.023
Std	0.012	0.013

## Discussion

This study analyzed the dependence of the GM on the PTV volume and shape (sphericity) for peripheral and central lung SBRT plans using the univariate and bivariate least-squares regressions. This study has demonstrated a predictable power equation between the GM of our center’s clinically acceptable lung SBRT plans and the PTV volume. The equational correlation coefficient of the peripheral lesions is higher than that of central lesions. Unexpectedly, the correlation between the GM and the PTV shape was very low for two types of lung SBRT plans. Overall, to the best of our knowledge, few studies were available on the shape dependence of dose gradient for lung SBRT plans. Moreover, few scholars classified and compared peripheral and central lesions in other similar research. This work defined the dependence of the gradient measure of lung SBRT plan on the PTV volume and shape. The results can predict the GM before planning and then set up the shell (pseudo structure) used for optimization individually to reduce possible GM increase and the number of trials and errors. In addition, the fitting equation obtained in this study can be used as a primary tool to evaluate the dose gradient after the planning process to assist the dosimetric quality control.

In a study by Hoffman et al. (7) on peripheral lung SBRT plans, although the factors of target shape and tumor type (peripheral or central) were not considered, their conclusions were similar to part results of this work. They also found that the dependence of the GM on the PTV volume presented a power relationship, and the functional form of that relationship was $\text{GM}=0.564{\text{V}}_{P}^{0.215}$ with a standard error of 0.017 and 0.006 for the two parameters and an R^2^ value of 0.850. The difference in R^2^ may be caused by data differences from two centers, such as the volume and range of the PTV, the treatment machine, the planning techniques, TPS, optimization methods, algorithms, etc. However, both we and Hoffman et al. found that the power function is the best to explain the relationship between the PTV volume and dose gradient. They got a slightly smaller GM (steeper dose fall-off) than this study (Eq. 8), which may be due to the data from different centers. We recalculated the GM of our enrolled cases using the results of Hoffman et al.’s study (Eq. 11), finding that the maximum and average analytic GM differences between the two studies were 0.10 and 0.063 cm, respectively. This shows that our results are almost consistent with those of Hoffman et al. Some other studies have also concluded a positive correlation between dose gradient and target volume (7, 13, 14), which agrees with our results.

The dependence of the GM on V_P_ for peripheral lesions showed a higher R^2^ than that for central lesions (0.675 vs. 0.527), indicating that compared with central lesions, the power function can better explain peripheral lesions. In other words, the GM of peripheral lesions has higher dependence on V_P_ than that of central lesions. This is probably because the positional relationship between the target and OARs is more complex for central lesions. In order to meet the dosimetric constraints, the planning parameters (such as the beam settings) of the central lung cancer are more diversified, which reduced the regularity of the dose gradient, leading to low dependence of the GM on the PTV volume. In addition, 7 of the 307 peripheral lung SBRT plans had a PTV volume greater than 125 cm ^3^. The residual (**Figure 2A_2_**) shows that when the PTV volume is greater than 125 cm^3^, Equation 8 for peripheral lung cancer will predict a higher GM, which is a limitation of the fitting results.

This study also investigated the dependence of the GM on the target shape (**Table 1**). However, for peripheral and central lung cancer, there were low correlation coefficients of the GM on shape variable Sp for all functional forms (R^2^ < 0.25), and the corresponding equations were not acceptable. Similar to the univariate results, the power-function form of the GM on bivariaty also got the highest R^2^ (**Table 2**). However, compared with the results from the univariate analysis V_P_, the R^2^ of bivariate results was not improved for two types of lung cancer. It demonstrates that the bivariaty (V_P_ and Sp) equations have the same explanatory power to the response variable GM as that of univariaty (V_P_). Moreover, the p values of Sp in the bivariate power functions were greater than 0.05, which indicates that the contribution of Sp to the equation is unreliable. Therefore, the bivariate equations serve no practical purpose. All those results proved that the GM has no dependence on the PTV shape.

There are few studies on the dependence of dose gradient on the target shape for lung SBRT plans, and the relationship between them has not been determined. This study provides definitive evidence proving that the dose gradient has no dependence on the target shape, which may be explained as the target volume suitable for SBRT is usually small, resulting in small shape ranges. Moreover, with radiotherapy technology advances, targets with different shapes can easily achieve high conformity in SBRT plans. These factors make the shape have little effect on the dose gradient.

Overall, only the power equation of GM versus V_P_ is reliable and acceptable for two types of lung cancer. The validation results using independent external data show that the mean absolute error of the GM for peripheral and central lung SBRT was less than 0.2 and 0.3 mm, respectively, which indicates that the GM’s final fitting formula is relatively reliable.

**Figure 4:**
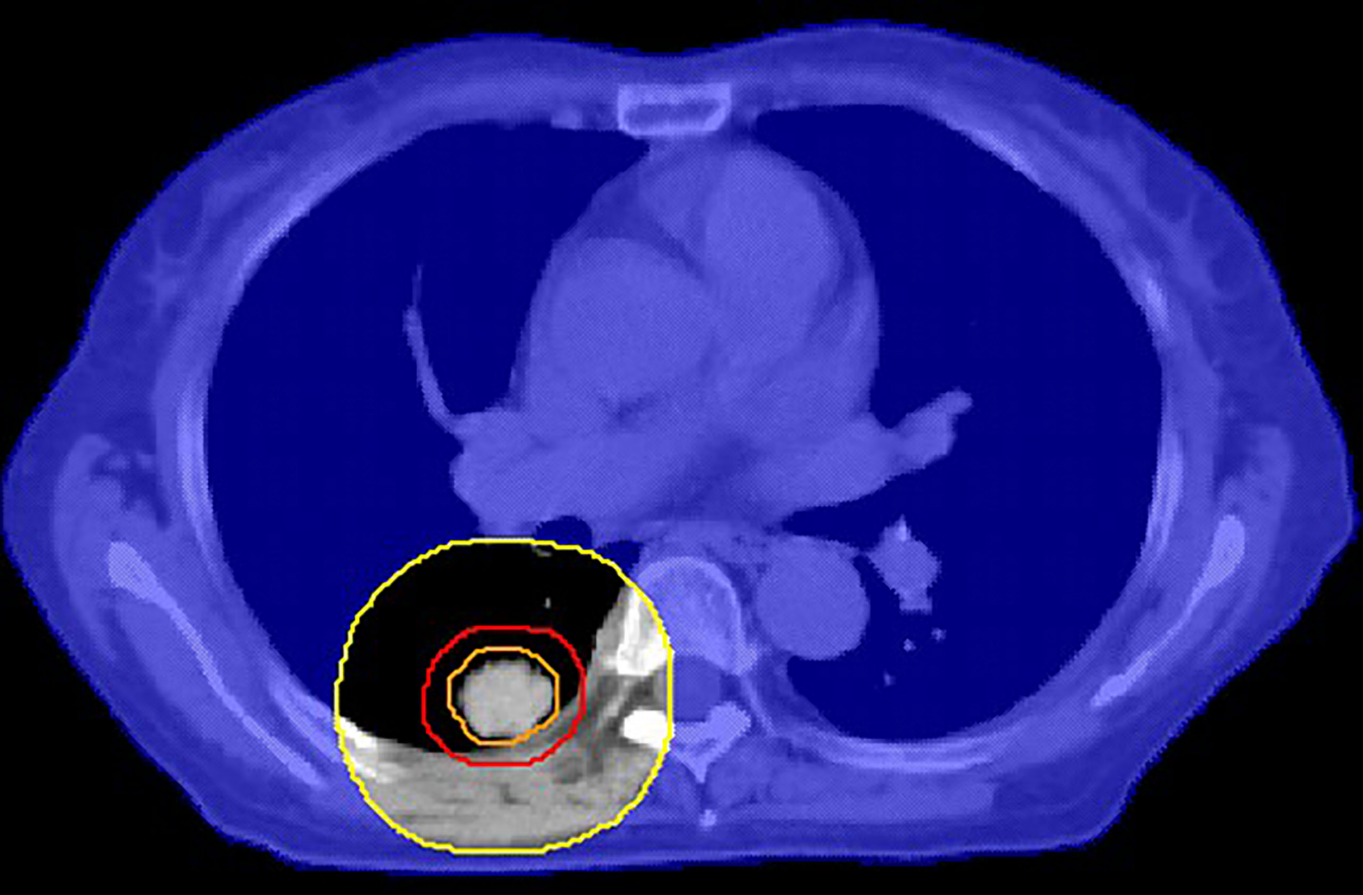
An example of a cross-sectional view of the shell (orange: ITV, red: PTV, yellow: auxiliary structure after GM cm expansion of PTV, blue shade: GM cm shell obtained by body minus auxiliary structure). This shell is used to control the 50% isodose line.

The results of this work can be applied to create a shell (pseudo-structure) to minimize the GM and hence achieve a sharper dose gradient during the clinical planning process. Since the CI is near unity for most SBRT treatment plans at our center, the average distance from the 50% isodose line to the edge of the PTV is approximately the GM. The results of this study can be used to develop the following possible workflow: 1) The GM is calculated using Equation 8 or 9 individually for patients using a prewritten script; 2) The planner creates a shell (15) (see **Figure 4** for details) with a distance of GM cm from the edge of PTV; 3) Before the optimization process, the maximum dose of the shell is set to lower than 50% prescription dose, such as 40%, to reduce the GM as much as possible; 4) After the preliminary plan, the planner benchmarks the plan against the GM. As part of plan quality control (QC), the shell from Equation 8 or 9 may be used to determine how the plan performed relative to the plans in the dataset. If the 50% isodose radius exceeds the shell, the plan may need to be adjusted by changing the constraint of the shell to achieve a possible lower GM. Naturally, the plan should finally meet the RTOG 0813 and RTOG 0915 dosimetric constraints.

It should be noted that, for safety reasons, the treatment of patients whose OARs in the SBRT plan do not meet the RTOG 0813 and 0915 guidelines will not be allowed in our center, but the alternatives will be considered. In other institutions, there may be other solutions to this situation. The guidelines of RTOG 0813 (16) and RTOG 0915 (17) recommend that the maximum dose at 2 cm from PTV in any direction should range from 50% to 77% of the prescription dose according to different PTV volumes. In this study, the analytic distances between 50% isodose lines and the PTV of the total 426 enrolled cases obtained by Equations 8 and 9 were less than 2 cm, which indicates that the analytic dose gradient is a more stringent constrain than D_2cm_ (Gy), which is suggested in the RTOG proposal and clinically achievable. In clinical practice, when the plan cannot meet the analytic results of Equations 8 and 9, doctors and planners should comprehensively evaluate the plan according to the guidelines such as RTOG and decide whether the plan can be applied to clinical treatment.

Here are some limitations and prospects of this study. This study did not include the impact of techniques (3D-CRT, IMRT, Arc), the number of beams, prescription dose, etc., on dose gradients. The impact of these factors on the results of this study requires additional data to explore (14, 20). In addition, the delivery machine used for this study was the Edge accelerator. The conclusion may be different if other equipment is used. The GM and target geometry relationship on other machines must be reexamined using methods similar to this study or other measures based on specific system characteristics. Finally, the obtained Equation 9 will overestimate the GM of the peripheral lung SBRT plan with PTV greater than 125 cm^3^. A separate equation could fit the large PTV data, but this requires more treatment plans in this volume range.

## Conclusion

The gradient measure of the lung SBRT plan is shape-independent but volume-dependent. The dependence of the GM on the PTV volume for peripheral and central lung cancer can be described by two different power equations, and the correlation is higher for peripheral lesions. The results of this study can be used for preplan prediction and postplan review of gradient measure and serve as a potential tool to assist dosimetric quality control during the radiosurgery process.

## Data Availability Statement

The raw data supporting the conclusions of this article will be made available by the authors, without undue reservation.

## Ethics Statement

The approved lung SBRT treatment plans in Shanghai Chest Hospital were retrospectively selected. When the study began, all selected patients signed informed consents and completed radiotherapy. Ethical standards and patients’ confidentiality were ensured and in line with regulations of the local institutional review board and data safety laws. This study was approved by the Ethics Committee of Shanghai Chest Hospital (the committee’s reference Number: KS1863).

## Author Contributions

YD: Study concepts, study design, data acquisition, statistical analysis, manuscript preparation, and manuscript editing. YL:Data acquisition, quality control of data and algorithms, and data analysis and interpretation. HW: Study design, statistical analysis, and manuscript review. BK: Quality control of data and algorithms and data analysis and interpretation. AF: Study concepts and manuscript review. KM: Quality control of data and algorithms. HC, YH, HG, and YS: Manuscript review. TZ: Study concepts and manuscript review. QK: Study concepts, study design, manuscript review, and wrote the paper. ZX: Study concepts, study design, and manuscript review. All authors contributed to the article and approved the submitted version.

## Funding

The authors thank the Nurture projects for basic research of Shanghai Chest Hospital (No. 2019YNJCM05) for its financial support.

## Conflict of Interest

BK was employed by MIM Software Inc., and KM was employed by Varian Medical Systems.

The remaining authors declare that the research was conducted in the absence of any commercial or financial relationships that could be construed as a potential confict of interest.

The authors declare that this study received funding from Nurture projects for basic research of Shanghai Chest Hospital (No. 2019YNJCM05). The funder was not involved in the study design, collection, analysis, and interpretation of data, the writing of this article or the decision to submit it for publication.

## Publisher’s Note

All claims expressed in this article are solely those of the authors and do not necessarily represent those of their affiliated organizations, or those of the publisher, the editors and the reviewers. Any product that may be evaluated in this article, or claim that may be made by its manufacturer, is not guaranteed or endorsed by the publisher.
